# *In silico* design of immunogenic antigen cocktail via affinity maturation-guided optimization

**DOI:** 10.1093/bioadv/vbaf182

**Published:** 2025-07-28

**Authors:** A N M Nafiz Abeer, Bong-Seong Koo, Byung-Jun Yoon

**Affiliations:** Department of Electrical and Computer Engineering, Texas A&M University, College Station, TX 77843, United States; Koreavaccine Co., Ltd, Seoul 04778, Republic of Korea; Department of Electrical and Computer Engineering, Texas A&M University, College Station, TX 77843, United States; Computing and Data Sciences, Brookhaven National Laboratory, Upton, NY 11973, United States

## Abstract

**Summary:**

The increasing emergence of new virus strains with increased infectiousness necessitates a more proactive approach for effective vaccine design. To achieve this goal, it is critical to shift the vaccine design paradigm from traditional approaches that rely on expert intuition and experimental methods toward data-driven strategies that leverage *in silico* design and virtual screening. In this work, we propose a computational pipeline for designing an optimized immunogenic cocktail that can boost the immune response. The proposed pipeline consists of two stages, where potential antigen candidates are identified in the first stage, followed by the optimal selection and combination of the candidates in the second stage to maximize the expected immunogenicity. We leverage predictive models trained using deep mutational scanning data to drive the candidate antigen selection process based on three selection criteria—namely, binding affinity between viral protein and receptor, antibody escape probability, and sequence diversity. To identify the optimal cocktail within the pool of selected antigens, we adopt a combinatorial optimization framework, where the cocktail design is iteratively refined based on the expected efficacy predicted by a sequence-based computational model of affinity maturation. Validation of the designed cocktails through structure-based affinity maturation simulation demonstrates the efficacy of the proposed modular framework for designing an optimized immunogenic cocktail.

**Availability and implementation:**

The code for cocktail design is available in https://github.com/nafizabeer/Antigen_Cocktail_Design.

## 1 Introduction

Demanded by the worldwide impacts of the coronavirus disease 2019 (COVID-19) pandemic, there have been accelerated efforts in designing robust vaccines for wider coverage breadth (Barouch 2022, Cankat *et al.* 2024). While most of the prominent approaches often opt to target a narrow range of circulating variants, the emergence of new variants poses a threat to the designed vaccines by escaping the neutralizing antibodies. For example, the study from Cao *et al.* (2022b) shows that several Omicron sublineages have mutations that enable them to evade the antibodies generated from the BA.1-related vaccine or infection. Experiments by Cao *et al.* (2023) demonstrate that the increased antibody escaping capability of the emerging variants is due to the convergence in mutational space guided by the imprinted humoral immunity. The mutations in the variants can also be associated with the increased virus-receptor interaction, e.g. binding affinity between angiotensin-converting enzyme 2 (ACE2) and receptor binding domain (RBD) of severe acute respiratory syndrome coronavirus 2 (SARS-CoV-2) (Martin *et al.* 2021). Furthermore, there is a risk of potential zoonotic spillover (Plowright *et al.* 2017, Sánchez *et al.* 2022) resulting in newer members to consider under the viral family. Given these possible driving factors of emergence, one would prefer a universal vaccine that can assist the immune system in fighting against all members of a certain viral family, requiring a much broader coverage breadth. In the case of the coronavirus family (*Coronaviridae*), the target or endpoint of this daunting task is the pancoronavirus vaccine (Cankat *et al.* 2024). The focus of our work is a subset of this bigger problem—designing vaccines for all variants, i.e. pan-variant vaccines. Specifically, we have demonstrated our framework for generating an immunogenic cocktail against potential variants of SARS-CoV-2.

Multiple works have adopted diverse strategies for designing vaccines to provide coverage against new variants of SARS-CoV-2. Nathan *et al.* (2021) focused on T-cell vaccines instead of B-cell inducing vaccines to fight the challenge of antibody evasive variants. Vishwanath *et al.* (2023) employed a combination of phylogenetic analysis and mAbs (monoclonal antibodies) specific mutations to design a pool of RBD sequences which was further narrowed down to the final antigen through *in vivo* screening. Some works (Zhang *et al.* 2023, Bruun *et al.* 2024) target the particular regions of RBD that are conserved across multiple variants and attempt to shift immune response toward them through the immunofocusing approach (Musunuri *et al.* 2024). Bruun *et al.* (2024) applied PMD (protect-modify-deprotect) technique (Weidenbacher and Kim 2019) to make the immune response more biased to RBD cryptic face which typically has subdominant response otherwise. Similarly in the works by Zhang *et al.* (2023), the glycan repositioning technique (Huang *et al.* 2022, Musunuri *et al.* 2024) in RBD resulted in an increased immune response toward targeted epitopes. Wang and Chakraborty (2022) utilized the deep mutational scanning (DMS) (Starr *et al.* 2020) experimental data for wildtype (WT) of SARS-CoV-2 along with conservation analysis to identify a set of single mutations within the RBD of the spike protein. Following some heuristics—maximal separation between mutations in the antigen sequences, diverse mutations based on amino acid types—they (Wang and Chakraborty 2022) formed six antigen sequences and performed an agent-based simulation of affinity maturation process (Shaffer *et al.* 2016) to compare the efficacy of different hand-picked combinations of those antigens.

The experiment of DMS (Fowler and Fields 2014) quantifies the effect of mutations (which can be single or multiple) on a reference sequence (e.g. WT) in terms of the functionality of interest such as stability, binding affinity, antibody escaping ability, etc. In the study by Wang and Chakraborty (2022), the mutations in antigen sequences were selected based on single mutation DMS data without considering the multiple mutation effects such as epistatic shifts (Starr *et al.* 2022) on the designed antigens. Our approach followed a screening pipeline for antigens with multiple mutations utilizing the prediction models trained with DMS experimental data for multimutant RBDs. Furthermore, finding the appropriate combination of antigens with the best efficacy requires an efficient search in a combinatorially large space, e.g. $\left(\begin{array}{c} N\\ 3 \end{array}\right)$ possible combinations for a cocktail of three out of *N* antigens. We proposed a combinatorial optimization stage that searched for the optimal immunogenic cocktail by maximizing the efficacy simulated by a computational affinity maturation process (Shaffer *et al.* 2016, Wang and Chakraborty 2022).

We have provided a summary of our methodology in Section 2.1. Specifically, Sections 2.2 to 2.4 describe three components of the framework—candidate antigen generation, cocktail design, and validation, respectively. Our contributions can be summarized as follows:

- We proposed a screening and optimization based framework for designing immunogenic cocktails to provide broader coverage against future viral strains.
- We generated a pool of candidate antigens utilizing prediction models developed with the DMS data of virus-receptor binding and antibody escape. This was further refined through clustering based on antigen sequence representation from a protein language model (pLM) to ensure high sequence diversity in the designed cocktail.
- We leveraged the cocktail efficacy feedback from affinity maturation simulation to guide the optimization strategies in the search for the optimal combination of antigens.
- We performed a structure-based affinity maturation simulation to investigate the immunogenic response of designed cocktails.

## 2 Methodology

### 2.1 Overview of the proposed framework

We aim to design a cocktail of antigens to make the immune system equipped with protection against potential new variants. This involves the identification of candidate antigen sequences from a reference sequence followed by a decision-making process to find the combination of antigens leading to optimum efficacy. Mutations within the new variants are unlikely to make RBD unstable or weaken its capacity to bind with ACE2 because either of these two can negatively affect the viral efficacy. To make our candidate antigens similar to potential new variants, we first select a set of single mutations on the basis of their effect on ACE2-RBD binding and RBD stability based on the deep mutational experiments with the reference sequence, i.e. the WT sequence. From the possible combination of these selected mutations, we generate sequences with double mutations (with respect to WT) and remove any ACE2-RBD nonbinding sequences predicted by an ensemble of neural networks. The remaining sequences are the candidate sequences with double mutations. Each of these sequences is mutated using the set of selected single mutations and the resulting set of sequences with three mutations is screened with the ACE2-RBD binding predictor. Those selected sequences again enter this process of mutation followed by ACE2-RBD screening. We continue this way to get a pool of sequences with a predefined number of mutations, e.g. five mutations. Note that once a residue position is mutated, that specific position of the sequence is skipped in the consequent mutation stages.

We prioritize those RBD mutant sequences that are likely to abrogate the RBD’s binding capability with known antibodies. Our intuition is that the immune system is vulnerable to those sequences and our cocktail should make the immune system trained to recognize and defend against them. We rank all sequences according to their predicted antibody escape probabilities and select the top 1% sequences with high escape probabilities for the next stage. The set of these selected Ab-escaping sequences can be very large and consequently, the size of all possible combinations of three antigen sequences becomes too large and challenging for the optimization algorithm to explore. To address this issue, we construct clusters of these samples based on their sequence embedding from a pLM. From each cluster, we select a small number of samples based on their optimality in Ab-escape probabilities and ACE2-RBD binding scores. The resulting small set of sequences will be the candidate pool for the cocktail design algorithm of the next stage. We follow such a cluster-guided random sampling approach to create a higher sequence diversity among the antigens in the design space of the cocktail optimization algorithm.

We formulate the cocktail design task as a combinatorial optimization problem of selecting the best combination of three antigens out of the candidate antigen pool processed in the previous stage. The optimization algorithm [Bayesian optimization and genetic algorithm (GA) in our work] seeks the optimum combination by optimizing the performance of the cocktail against a panel of test antigens. To compare the antibody growth among different combinations of antigens, we run a sequence-based computational model that simulates the affinity maturation process for the cocktail. After the end of the maturation process, we consider the mean titer count, i.e. resulting number of B-cells with high affinity to the test antigens as the efficacy of the cocktail. This affinity maturation model makes several assumptions making it very computationally cheap. However, it also makes the solution found by the optimization algorithm less reliable. Therefore, we further validate the designed cocktails by investigating whether they demonstrate expected affinity maturation dynamics in a separate 3D structure-based computational model.

**Figure 1.**
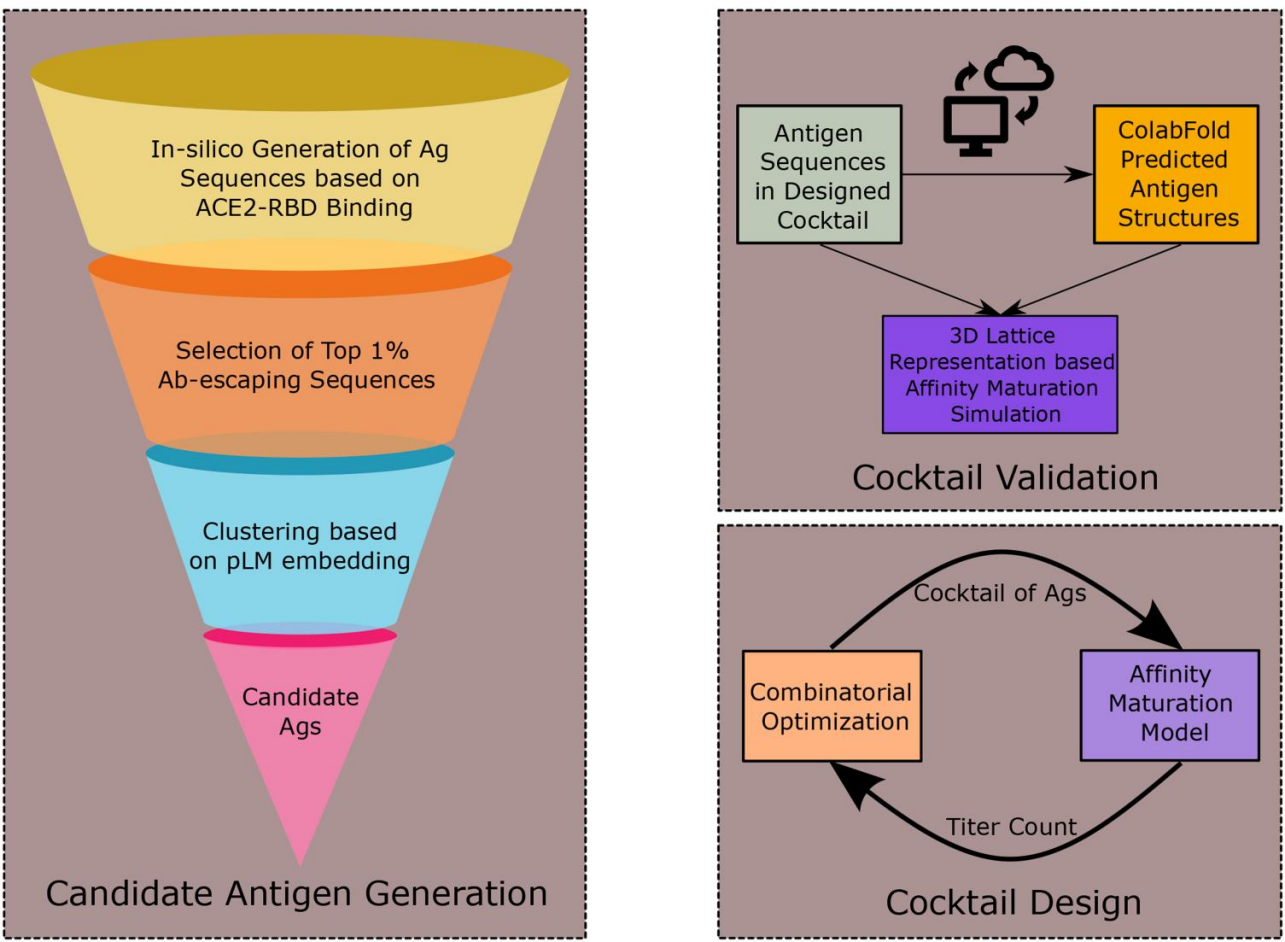
*In silico* design and validation of antigen cocktail. The framework consists of three components: candidate antigen generation, cocktail design, and validation. Candidate antigen generation—the candidate Ags are generated based on ACE2-RBD binding, Ab-escape probabilities, and sequence diversity. The first two criteria are dictated by predictor networks trained with DMS data while the latter is achieved implicitly by clustering of antigen sequence embeddings from a pLM. Cocktail design—the iterative optimization of cocktail is guided by efficacy feedback from a sequence-based AM model. Cocktail validation—the immunogenic response of designed cocktails are validated via more complex computational model of AM which utilizes the structural information of antigens.

Figure 1 summarizes the major components of our approach to cocktail design: generation of candidate antigens pool, followed by cocktail design and validation. Within the scope of this article, the following subsections provide the details of the methodologies that we have used in each component.

### 2.2 Candidate antigen generation

Our design expectation is to have a cocktail of antigens with enough variability so that the immune system gets the experience to defend against the newer variants. To achieve that, we focus on generating a pool of antigens with a certain number of mutations with respect to a reference sequence. We can use any sequence as the reference given that we have access to the data from deep mutational experiments (Fowler and Fields 2014) with that sequence. For this work, we consider the WT of SARS-CoV-2 spike protein’s RBD sequence. Below we describe how we create a large collection of mutant sequences starting from WT and select a small subset of that collection to be used as potential candidates in the cocktail design stage.

#### 2.2.1 *In silico* generation of antigen sequences

For protection against the potential variants, each antigen of the cocktail should have similarities with those variants in terms of its mutation. This becomes a forecast problem for the future mutations on the WT. At the early stage of a viral outbreak, this is a challenging task for a data-driven algorithm due to the very small sample size of mutant sequences. Keeping this concern in mind, we make an effort to use data from the WT sequence-related experiments in almost every stage, except the antibody escape probability prediction. Consequently, we follow a few heuristics to generate possible mutant sequences instead of pursuing the generative techniques involving different pLMs. (Rives *et al.* 2021, Rao *et al.* 2021).

We generate the mutant sequences from a set of selected single mutations for the WT. This selection criterion is based on the hypothesis that the mutation of a sequence will not change the RBD stability beyond some extent compared to WT. Additionally, the mutation should not weaken the binding affinity between RBD and ACE2. DMS experiments reported by Starr *et al.* (2020) provide a quantitative comparison of the effects of all possible single mutations on RBD expression and the ACE2 affinity for WT. For every RBD residue, we have 19 possible amino acid substitutions and for each of these mutations, DMS experiment quantifies a relative change in RBD expression/ACE2 binding with respect to the WT sequence. For example, if the change is positive, the corresponding mutation will make the mutant sequence have a higher quantity of interest, e.g. increased binding affinity with RBD. We select only those mutations for which that change is above some value, $\delta_{0}$. With $\delta_{0}<0$, we allow some single mutations that negatively affect the RBD functionality. This decision is based on the mutational accumulation effect. Specifically, it is possible that a mutation at a single residue can affect the mutational effect at another position due to the epistasis (Starr *et al.* 2022). Consequently, any of those single mutations can appear in a sequence with multiple mutations, and their combined effect on the RBD stability and the ACE2-RBD binding affinity can be positive, relative to the WT. However, if we choose a very large negative value of $\delta_{0}$, then we end up with all 19 mutations for each residue leading to a huge computational burden. Therefore, we need to set this threshold to a value that gives us a large collection of mutations considering the computational resources. In this work, we set $\delta_{0}=-0.2$ as in Wang and Chakraborty (2022) which results in 219 single mutations within 38 RBD residues.

Given this set of selected single mutations, we can create sequences with two mutations by taking all possible combinations. Similarly, we could go on generating sequences with more mutations but that would create a large set of sequences where some of them are not in our interest. To avoid that, we identify whether those sequences with double mutations bind with ACE2 or not. Based on the binding prediction (Section 2.2.2), we keep only those mutant sequences that bind with ACE2. Now each of these sequences can again get mutated using the selected single mutations. By repeating this process, we can generate a large set of ACE2-binding RBD sequences with a fixed number of mutations. In Section 2.2.3, we describe how this large set can be pruned out by utilizing another experimental outcome from DMS, antibody escape fraction.

Since the ACE2-RBD predictor described in Section 2.2.2 is trained with sequences from the receptor-binding motif (RBM) regions of RBD, any mutation outside RBM regions does not have any impact on the prediction. Thus, effectively we have to select single mutations only within RBM residues. This constraint is not needed if the ACE2-RBD binding prediction network is trained with RBD sequences where mutations are distributed over all residues. Moreover, our approach only searches for the candidate antigens within the restricted search space spanned by the initial set of mutations which are selected based on the single-point mutation DMS data. When we consider multiple mutations in a designed sequence, our initial set may not necessarily include mutations which are more preferred in a multimutant sequence (e.g. based on pLM log-likelihood ratio). Hence, while a larger $\delta_{0}$ leads to lower computational requirements due to the smaller number of useful initial mutations, it also potentially limits our Ag design pipeline’s exploration within a suboptimal sequence space.

#### 2.2.2 ACE2-RBD binding predictor

Given an RBD sequence with multiple mutations, our prediction model needs to classify it as binding or nonbinding with ACE2. We formulate the classification task by considering three receptor binding motif (RBM) regions: RBM-1 (453–478), RBM-2 (484–505), and RBM-3 (439–452) because mutations within these core regions of RBD significantly affect the interface between RBD and ACE2. We build separate classifiers, $f_{i}$ for each RBMs and aggregate their output (Equation (1)) as the final classification label, $y_{\text{ACE}2}\in \{0,1,2,3\}$ for the RBD sequence.


(1) $$\begin{array}{c} y_{\text{ACE}2}=f_{\text{ACE}2}(x_{\text{RBD}})\\ =f_{1}(x_{\text{RBM}-1})+f_{2}(x_{\text{RBM}-2})+f_{3}(x_{\text{RBM}-3}). \end{array}$$

Each RBM-specific predictor is an ensemble of two networks of different architecture: LSTM and transformer. The one-hot representation of the RBM sequence is fed to each network separately. For the LSTM network, the hidden state for the last token of the input sequence after $n_{\mathrm{LSTM}}$ layer goes through a fully connected network with two layers to produce the ACE2 binding probability. The transformer-based predictor first combines the one-hot embedding of the given RBM sequence with its positional encoding. Then a stack of $n_{\mathrm{enc}}$ encoder layers processes the resulting embedding with self-attention and feed-forward network. Finally, the prediction is formed as the output of a fully connected network that takes this encoded embedding of the input. When both LSTM and transformer-based models predict a given RBM subsequence to be binding with ACE2, then we assign the output label, $f_{i}(\cdot )$ to be 1, otherwise 0, i.e. we conclude that it is a nonbinding subsequence. Consequently, if we have $y_{\text{ACE}2}=0$ for an RBD sequence, we interpret the prediction as indicating that ACE2 does not bind to any of the RBM regions.

#### 2.2.3 Selection of candidate antigens

The number of mutant sequences generated based on ACE2-RBD binding can be very large as the majority of selected single mutations positively impact the ACE2 binding. Since our cocktail design stage involves solving a combinatorial problem of finding the best cocktail of available antigens, we reduce the number of these sequences in two steps. First, we prioritize those sequences that are likely to escape the neutralizing antibodies. Different studies (Cao *et al.* 2022a, Iketani *et al.* 2022) found that newer variants prefer mutations that result in immune evasion from antibodies. We consider 10 antibodies from Reincke *et al.* (2022) to create a rank among the mutant RBD sequences based on the predicted probability of escaping these antibodies. This Ab escape predictor (Wang 2023) is described in Supplementary Information, available as supplementary data at *Bioinformatics Advances* online. We considered these 10 antibodies (corresponding to the beta variant) because the Ab escape prediction model did not see them during its training. However, they can be replaced with any antibodies of interest. For each RBD sequence, we predict the escape probability for each of the 10 antibodies. Based on the average predicted escape probabilities, we keep only those antigens above the 99th percentile. We note that how these 10 antibodies might be related to the viral escape profiles of future variants and whether they are representative of the broad human antibody repertoire may affect the pool of top 1% Ab-escaping antigen sequences, and thus potentially lead to suboptimal search space for our cocktail optimization.

Since the antibody escape predictor tries to predict the output from the sequence space, similar sequences are likely to have close escape probability predictions. This potentially could lead to a high degree of sequence similarity among the top 1% Ab-escaping antigen sequences. To address this, we cluster these sequences with the help of a pretrained pLM. We first get the embedding of each residue of the antigen sequence from the ProtBert model (Elnaggar *et al.* 2021), and the average of RBD residue embeddings is defined as the sequence embedding of the corresponding antigen. This averaging may not be an optimal way of aggregating the residue level embedding (Vu *et al.* 2023) but it provides an effective summary of the whole sequence.

The high-dimensional sequence embedding is reduced by taking the projection on a smaller number of principal components. This minimum number of components explains 95% variance of the pLM’s embedding for all sequences generated based on the ACE2-RBD binding. Using the projected embedding, the top 1% sequences are clustered with the Bayesian Gaussian mixture model (GMM). From each cluster, we first find those sequences that are the most optimal (Pareto optimal to be specific) in terms of the ACE2 binding and the Ab-escape probability. Randomly selected two sequences from each of these Pareto optimal sets are considered as candidate antigens. Specifically, we find the Pareto optimal set of antigens in each cluster based on their predicted ACE2-RBD binding $y_{\text{ACE}2}$ and the Ab-escape probability $y_{\text{Ab-escape}}$. The Pareto optimal set for each cluster contains those antigens that are not dominated by other antigens in that cluster according to the following definition: antigen *i* dominates antigen *j* when we have $y_{\text{ACE}2}^{\left(i\right)}\;\geq \;y_{\text{ACE}2}^{\left(j\right)}$, $y_{\text{Ab-escape}}^{\left(i\right)}\;\geq \;y_{\text{Ab-escape}}^{\left(j\right)}$ and at least one of these relations holds with strict inequality. If a cluster has only one antigen in its Pareto optimal set, we move to the next Pareto optimal set for the rest of the antigens in that cluster. Compared to the weighted objective approach, this Pareto ranking approach is more robust to the trade-off between conflicting objectives, and has previously been demonstrated to be an effective multiobjective ranking scheme for small molecules in generative molecular design (Abeer *et al.* 2024).

We take Pareto optimal samples from each cluster to have an increased sequence diversity in the candidate pool. Any other heuristic is also applicable as long as the size of this pool is small enough to be effectively explored by the cocktail optimization algorithm. Furthermore, the embeddings derived from ProtBert-like pretrained pLMs may not reflect the evolutionary selection context originating from viral escape mutations since the pretraining procedure contains general protein sequences. One may adopt viral-family specific fine-tuning of the pretrained pLM to make the antigen clusters more representative of distinct escape characteristics.

### 2.3 Combinatorial design of cocktail from candidate antigens

We get the pool of candidate antigens by pruning out a larger collection based on ACE2-RBD binding, antibody escape probability and sequence diversity. Designing the optimum cocktail of three antigens from a pool of *N* candidates means finding out the best combination from $\left(\begin{array}{c} N\\ 3 \end{array}\right)$ possible ones. Even for a moderate value of *N*, it is computationally impractical to estimate the efficacy of all possible combinations exhaustively. Thus, we seek this cocktail design task through the lens of combinatorial optimization for efficient exploration of the large design space.

Let’s denote the cocktail as an *N* dimensional vector, $x\in {\left\{0,1\right\}}^{N}$, where ith element $x_{i}$ is 1 if ith antigen is present in the cocktail, otherwise it is zero. The order among these *N* antigens is arranged according to their cluster labels. For example, with N=20 the first two antigens are from the first cluster, the next two from the second cluster, and so on for the next eight clusters. We can formulate the design of a cocktail with three antigens as the following optimization problem:


(2) $$\underset{x\in {\left\{0,1\right\}}^{N}}{\max}\;f_{\text{AM}}(x)\;s.t.\;\sum_{i=1}^{N}x_{i}=3.$$

The optimization algorithm tunes the design variable x to maximize the efficacy of the corresponding cocktail. We define the efficacy of the cocktail, $f_{\text{AM}}(x)$ as the amount of antibodies generated with higher reactivity to a set of test antigens. A cocktail with a good combination of antigens is expected to produce antibodies that can protect against multiple test antigens of interest. A possible qualitative indicator of such efficacy is the number of B-cells having high affinity to those antigens. As we want the cocktail to be effective for the possible new variants, we select 1000 sequences randomly from the remaining top 1% Ab-escaping RBD sequences to form the panel of test antigens. We do not include any of the candidate Ags in this test panel to reduce bias toward those sequences during optimization. It is worth exploring further whether a more strategic selection, such as maximizing diversity within the test panel or mutation at structurally relevant residues, may offer a more effective broad efficacy measure.

We obtain $f_{\text{AM}}(x)$ by simulating the affinity maturation process triggered by the immunization with the cocktail represented by x. Due to the stochastic nature of simulation, we run the affinity maturation process for 10 times for a given combination of candidate antigens and the average size of the B-cell population with high affinity is used as the cocktail efficacy metric. We use the computational model from (Sprenger *et al.* 2020, Wang and Chakraborty 2022) to simulate the antibody development mechanism. In the Section 2.3.1, we describe how this computational model approximates the binding affinity from the antigen sequences. Further details about the dynamics of the germinal center (GC) reaction can be found in Wang and Chakraborty (2022). As for the optimization scheme, we investigate the performance of greedy optimization (Section 2.3.2), Bayesian optimization (Section 2.3.3), and GA (Section 2.3.4) in terms of their efficiency in discovering optimum cocktail.

#### 2.3.1 Computational model of affinity maturation

In the affinity maturation model from Wang and Chakraborty (2022), the antigen epitope is represented as a vector of 50 residues, where no actual order among the residues is considered. Within these 50 residues, we assign the first 38 positions for the single mutations we have identified at the beginning of the *in silico* generation of RBD sequences. This corresponds to the variable epitopes. The remaining 12 residues represent the conserved epitopes. Based on the design variable x, we extract the RBD residues at those 38 positions of the corresponding antigens. Then we find the antigen epitope vector for them according to the change in amino acid type with respect to the WT. First, we initialize the epitope vector with all ones. Following Wang and Chakraborty (2022), we go through the set of mutations of each antigen and assign the following values for the corresponding index in the vector.

−1 if the residues before and after the mutation belong to the same class of amino acid.

−2 when the change is between polar and charged or hydrophobic and polar.

−3 when mutation makes a hydrophobic amino acid to charged one or vice-versa.

−4 for transition between positive and negatively charged amino acids.

This epitope representation is then used to compute the binding energy between the B-cells and the antigens in the cocktail which influences the selection of the B-cells for mutation during the affinity maturation process. After the completion of GC reaction, activated upon the immunization by the cocktail, the binding energy between each B-cell and antigens from the test panel is computed. We count the number of B-cells for which the binding energy for a test antigen exceeds a threshold. The average of these counts over the size of the panel of test antigens is the titer count for this GC simulation. Finally, the mean value of the titer counts from multiple simulations quantifies the efficacy of the cocktail defined by x.

#### 2.3.2 Greedy optimization

In the greedy optimization scheme, we sequentially grow the cocktail size from one antigen to three antigens by greedily selecting one antigen at a time based on the expected efficacy of the resulting cocktail when the antigen is added to current design. Specifically, we first compute the efficacy for all *N* antigens individually. Next, we form a cocktail of one antigen by selecting the one with the best efficacy out of these *N* antigens. This means the cocktail x has all zero entries except for the position corresponding to the antigen. Next, we go through the remaining N−1 antigens to add another antigen to our current design x such that the cocktail of both antigens produces maximum efficacy. For the next step, we follow the same process for the remaining N−2 antigens to get our final design, i.e. a cocktail of three antigens.

Note that this sequential greedy approach limits our search to N−2 possible choices for a cocktail of 3 antigens instead of $\left(\begin{array}{c} N\\ 3 \end{array}\right)$. Although the one antigen selected in the first step is optimal for a cocktail of size one, it may not be optimal in the presence of another antigen in a cocktail of size two. Similarly, this can lead to suboptimality in the cocktail of three antigens. Hence, the greedy optimization approach may not find the optimal combination of three antigens. Furthermore, while this approach is computationally cheap (3(N−1) number of efficacy evaluations), we are not effectively exploring a diverse search space. In the next two sections, we describe Bayesian optimization and GA which employ different mechanisms for efficient exploration of the search space.

#### 2.3.3 Bayesian optimization (BO) via probabilistic reparametrization

We first collect a small number (five in our experiment) of feasible cocktails, i.e. satisfying the constraint of three antigens, and measure their performance using the computational model. A Gaussian process, $f_{\text{GP}}$ is trained to maximize the likelihood for these initial samples and their objective values. To suggest the next cocktail, we optimize the acquisition function which is valid in the discrete values of each $x_{i}$. For optimization over this discrete design variable, we follow the probabilistic reparameterization from Daulton *et al.* (2022). Instead of optimizing over the discrete x, we have a continuous design variable, $\theta \in {\left[0,1\right]}^{N}$. At each iteration, we optimize the acquisition function, α(x) by selecting the $\theta_{\text{next}}$ according to Equation (3).


(3) $$\theta_{\text{next}}=\text{arg}\;{\text{max}}_{\theta }E_{x∼p(\cdot |\theta )}\left[\alpha (x)\right].$$

(4) $$\alpha (x)=\max\left(f_{\text{GP}}(x)-f^{*},0\right)\times 1_{\sum x_{i}=3}.$$

We choose the constrained expected improvement as the acquisition function. In Equation (4), $f_{\text{GP}}$ is the Gaussian process model at the current iteration and $f^{*}$ is the highest efficacy among the candidate cocktails evaluated before the current iteration. $1_{\Sigma x_{i}=3}$ is an indicator function that produces 1 when the cocktail has only three antigens, otherwise it is 0.

To get the next candidate in the discrete domain, we sample $x_{\text{next}}$ from the conditional distribution, $p(\cdot |\theta_{\text{next}})$ which is *N* dimensional Bernoulli distribution with success probability $\theta_{\text{next}}$. We update the Gaussian process $f_{\text{GP}}$ with this candidate $x_{\text{next}}$ and its efficacy $f_{\text{AM}}(x_{\text{next}})$, and move to next iteration. This optimization process continues until we exhaust the allocated budget for evaluating the candidate cocktails’ efficacy.

#### 2.3.4 Genetic algorithm

The GA is initialized by randomly selecting $n_{\text{pop}}$ number of cocktails with three antigens as the initial generation of population. We use $n_{\text{pop}}=5$ to match the size of initial samples in Bayesian optimization. Two members from the current generation are randomly selected as parents with the corresponding selection probability being proportional to their objective values. The crossover between these parents produces two new solutions. Such a new cocktail is formed by taking one randomly selected antigen from one parent and the remaining two from the other parent. These two new cocktails as well as the current generation of the population go through mutation to accelerate the search for the optimal solution. With a mutation rate, $r_{\text{mut}}$ a cocktail is selected for mutation and one of its three antigens is randomly substituted by any of the remaining N−3 candidate antigens. Note this mutation process of the GA does not alter the Ags’ residues. After these mutations, the new cocktails are added to the current population. To keep a fixed size of the population over all generations, only the top $n_{\text{pop}}$ unique cocktails with high efficacy are considered as the next generation’s population.

We run this iterative process for a fixed number of steps which closely matches the number of objective function queries for the Bayesian optimization. We found that the number of unique queries for the efficacy of the cocktail is largely dependent on the mutation rate. At each generation, the GA produces two new cocktails. Sometimes it is possible that these two cocktails may correspond to a cocktail we have previously considered. In such cases, we took the previous evaluation rather than running the computational model again.

### 2.4 Validation of designed cocktails

The efficacy obtained from the affinity maturation simulation in Section 2.3.1 cannot correctly reflect the underlying complex interaction involved in the antigen and the B-cell receptors. As the antigen-antibody binding is computed from a simple representation of antigen epitope vector, the role of structure of the antigen is sacrificed in favor of the fast estimation of efficacy. Thus, the designed cocktail by the optimization algorithm may not correspond to an effective cocktail. Robert *et al.* (2024) shows that the presence of a strong immunogenic antigen can dominate the GC reaction by inhibiting the affinity maturation of less immunogenic antigen in the cocktail. In such cases, the immune system primarily creates antibodies for the dominant antigen. For our designed cocktail, we want a balanced immunogenic response to all three antigens in the cocktail since we are using it to prompt the immune system to respond to different antigens, not just a single antigen.

To investigate whether we have such an imbalanced GC response to a single dose of our designed cocktails, we use the vaccine simulation from Robert *et al.* (2021) where the 3D antigen structure is used to calculate the antigen-antibody affinities. We run the simulation for a designed cocktails with the same set of parameter values as in Robert *et al.* (2024). Since the cocktails contain RBD sequences with multiple mutations, we decided not to use the structure of the WT sequence. Instead, we predicted the structures of each antigen with ColabFold (Mirdita *et al.* 2022). These predicted structures are further processed to the corresponding 3D lattice via Lafit tool (Mann *et al.* 2012). The simulation utilizes this lattice representation of the antigen structure and the complete RBD sequence for approximation of the binding energy between the antigen and antibody (the CDRH3 region of the heavy chain to be specific). Robert *et al.* (2021) integrates this structurally derived affinity into the computational method for GC reaction from Meyer-Hermann *et al.* (2012). This makes the coarse-grained simulation more realistic compared to the one we use during the optimization stage.

## 3 Results and discussion

### 3.1 Training of ACE2-RBD predictor

We train six networks (two architectures, three RBM regions) separately with the SARS-CoV-2 RBD variants dataset from a study by Taft *et al.* (2022). Specifically, we used the ACE2 binding screening result for the combinatorial RBD mutagenesis library focusing on three RBM regions. We split each RBM’s data for ACE2 binding activity into training (80%), validation (10%), and test (10%) dataset. For each network, we applied the Optuna (Akiba *et al.* 2019) to tune a few hyperparameters, e.g. number of LSTM/transformer encoder layers, dropout probability, etc. by optimizing the average precision on the validation split. We used the *average_precision_score* from Scikit-learn (Pedregosa *et al.* 2011) on the predicted binding probabilities from the network and the true class label to compute the mean precision score. Details of this procedure are given in Supplementary Information, available as supplementary data at *Bioinformatics Advances* online.

Table 1 compares the performance of using each architecture against their combination in terms of the accuracy and precision on the held-out test dataset. The increase in precision by making use of both predictors is significant for our work since we use the predictions to screen a large collection of mutant sequences for the ACE2-binding sequences and we want a higher percentage of those selected sequences to be true positive cases.

**Table 1.** Test accuracy and precision for the LSTM and Transformer-based separate and consensus prediction.^a^

Binding region	Test accuracy			Test precision		
	Transformer	LSTM	Consensus	Transformer	LSTM	Consensus
ACE2-RBM1	0.9194	0.9460	0.9403	0.9070	0.9537	0.9622
ACE2-RBM2	0.9189	0.9330	0.9306	0.9157	0.9455	0.9510
ACE2-RBM3	0.9506	0.9569	0.9543	0.9474	0.9570	0.9605

a Consensus prediction is obtained based on the agreement in the binary output of LSTM and Transformer-based networks. Accuracy is the fraction of test data points with correct predictions. Precision is defined as the fraction of true binding sequences among predicted binding cases.

### 3.2 Candidate antigens show the desired high sequence diversity

Based on the procedure described in Section 2.2.1, we first identified 219 single mutations, and iteratively enlarged the pool to include sequences with five mutations (selected from those 219 single mutations) with respect to the WT sequence. At each stage, we had a filter to remove any ACE2 non-binding mutant sequence, i.e. $y_{\text{ACE}2}=0$ predicted by the consensus network described in Section 2.2.2. We computed the sequence embeddings for this pool of remaining sequences (1 975 187 in total) using the ProtBert model (Elnaggar *et al.* 2021). These 1024 dimensional sequence embeddings were further projected to a low-dimensional space (19 in our case) via principal component analysis (PCA) based on the 95% explained variance criterion. Next, we selected the top 1% of the pool based on their predicted Ab-escape probabilities, and learned a GMM to cluster them based on their low-dimensional embeddings. For each of the 10 clusters, the Pareto optimal sequences were identified based on predicted ACE2-RBD binding and Ab-escape probability and we randomly selected two samples as antigen candidates from these optimal sequences. These procedures led to a candidate pool of 20 antigens (each with five mutations with respect to WT) for the cocktail optimization stage.

**Figure 2.**
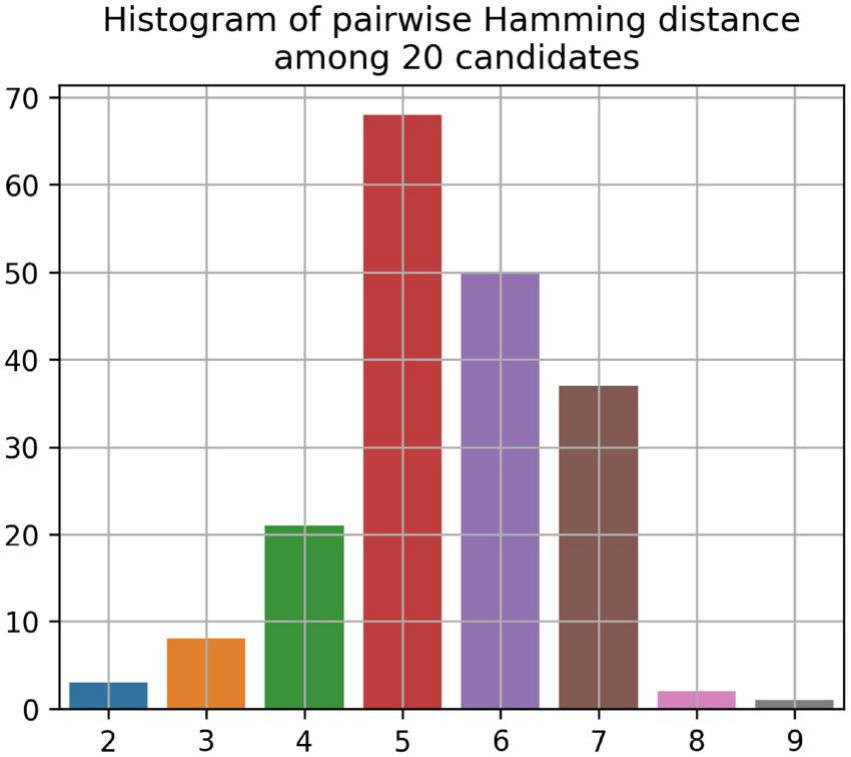
Sequence diversity of 20 candidate antigens. Histogram is generated based on the Hamming distances among unique pairs (190 pairs) of 20 selected antigens for cocktail optimization.

Figure 2 shows the sequence diversity (in terms of the Hamming distance) among the selected 20 antigen candidates. The histogram of 190 unique pair distances indicates that the candidate antigens for the cocktail optimization task have high diversity which may help provide broad coverage against potential new variants. However, this high diversity can also lead to unexpected dynamics (e.g. immunodominance) in the affinity maturation process warranting further validation.

### 3.3 Combinatorial optimization effectively finds out cocktail with high efficacy

Given the 20 candidate antigens, we applied three different optimization techniques—greedy optimization, Bayesian optimization, and GA—to solve the problem in Equation (2), i.e. designing an optimal combination of three antigens using the efficacy feedback from affinity maturation model of Section 2.3.1. Table 2 shows the sequential progression of the cocktail design under the greedy optimization approach where the final cocktail of 3 antigens is (3,4,20).

**Table 2.** Sequential progression of greedy optimization.^a^

Number of Ags in cocktail	Ag IDs in greedily selected cocktail	Efficacy $f_{\text{AM}}(x)$
1	4	3524.73
2	3, 4	4363.36
3	3, 4, 20	4325.68

a Greedy optimization procedure sequentially goes from a cocktail of 1 to 3 antigens. The Ag IDs for the cocktail design at each stage represent which antigens out of 20 candidate antigens are present in the cocktail.

**Figure 3.**
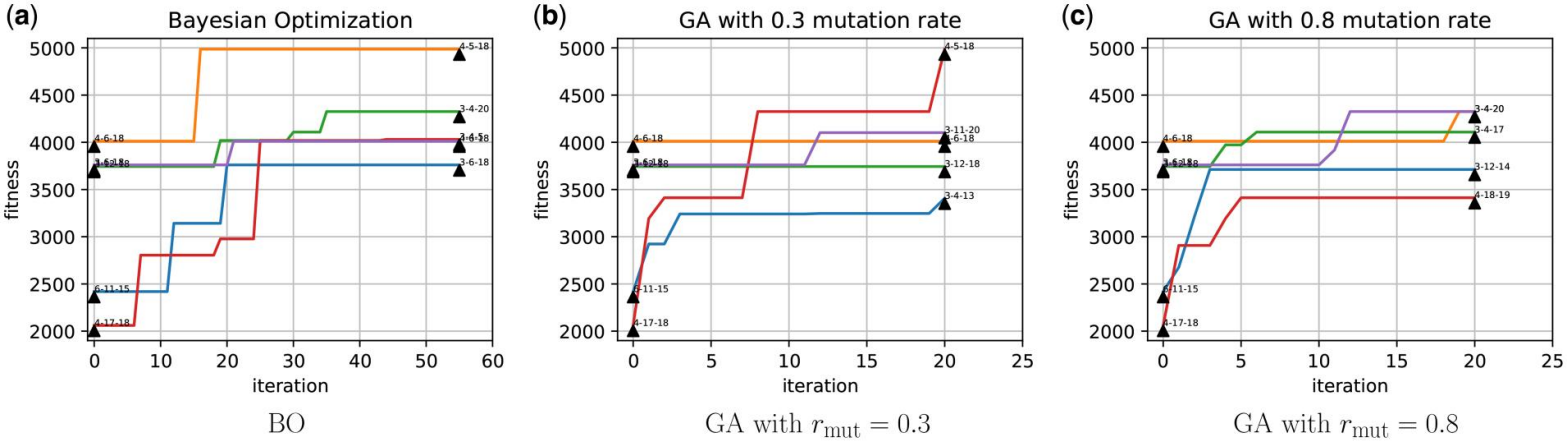
Optimization traces for affinity maturation guided cocktail design. For Bayesian optimization (a) and genetic algorithm (b, c), traces for five trials show the best fitness (efficacy) value found at each iteration. The fitness at 0^th^ iteration corresponds to the best cocktail out of five randomly sampled cocktails (used to initialize the optimization strategy). We also show the antigens present in the best cocktail at the 0^th^ and final iteration. For example, 4-5-18 denotes the antigen IDs of the best cocktail at the corresponding iteration.

For Bayesian optimization and GA, we started from a pool of five cocktails derived from randomly selected three antigens out of 20 candidate antigens. Hence, the initial best cocktail (out of the pool of five cocktails before any optimization) is identical for both strategies leading to a fair comparison among them. We repeated the optimization 5× times (using different random seeds) for each technique, and Fig. 3 shows the corresponding optimization traces where the fitness is the titer count for the designed cocktail simulated by the computational model of affinity maturation. Each subfigure shows the best cocktail at the 0th iteration (before optimization) and final iteration for all five trials. Figure 3b and c show the results with GA described in Section 2.3.4 with two different mutation rates, i.e. $r_{\text{mut}}=0.3,0.8$. Note that GA with higher mutation rate can explore larger number of cocktails in 20 iterations compared to Bayesian optimization (Section 2.3.3) via Probabilistic Reparametrization) with 55 iterations (Fig. 3a). For all five trials, the Bayesian optimization effectively improved the cocktail’s design. On the other hand, the GA with both mutation rates struggled to improve when starting from a cocktail with relatively good fitness value. Specifically, we observed this scenario for traces: orange, purple and green in Fig. 3b and c. Unlike Bayesian optimization which tries to solve the global optimization by learning a surrogate model, the GA’s capacity is limited by the current knowledge about best candidates making it more prone to get stuck at a local optimum solution.

In one of the five trials, both Bayesian optimization and GA (with a lower mutation rate) are able to find a cocktail (4,5,18) with higher efficacy that outperforms the cocktail (3,4,20) found by the greedy optimization approach. Even for the other four trials, the Bayesian optimization shows quite effective exploration in finding cocktails with improved efficacy, especially when we initialize the optimization with very weak-performing cocktails. While the performance of both Bayesian optimization and the GA partly depends on the cocktails used for initialization of the corresponding algorithms, they enable the exploration of more diverse search space than the myopic strategy of the greedy optimization approach.

### 3.4 *In silico* validation of designed cocktail

The cocktail optimization process utilizes the computational model of affinity maturation (Section 2.3.1) which considers only the type of mutation with respect to WT sequence. This simpler model does not reflect more complex dynamics such as the immunodominance relation (Robert *et al.* 2024) among the antigens present in the cocktail. So, we validated our designed cocktail with the structure-based affinity maturation model of Robert *et al.* (2021). Out of the different cocktails found during the combinatorial optimization process in Section 3.3, we have considered the top two and the worst (among the best cocktails found across different iterations of optimization) cocktails for validation. Although this *in silico* investigation cannot evaluate the cocktails in an absolute manner, it can provide a comparison among the GC’s responses to the cocktails.

**Figure 4.**
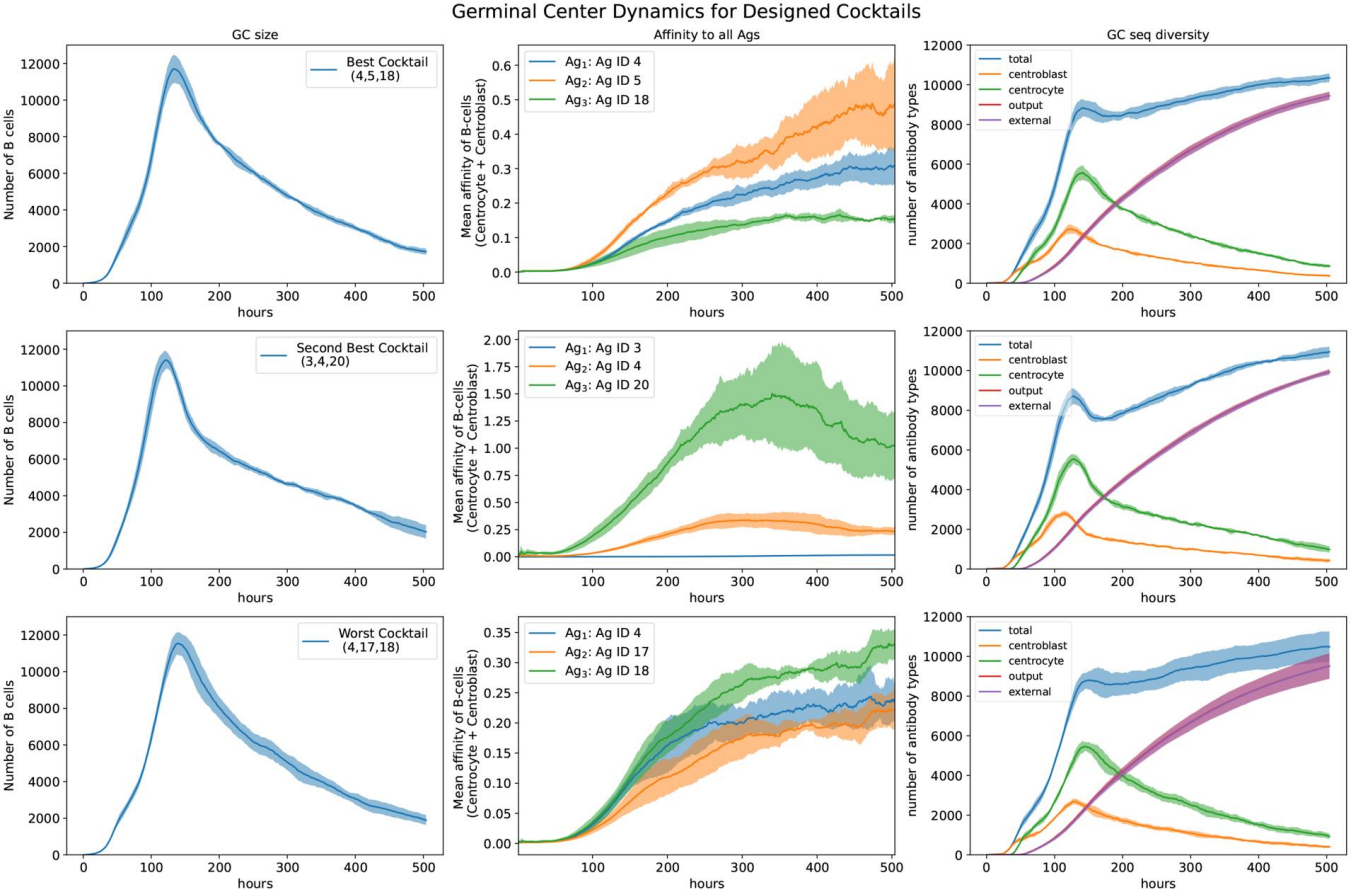
Germinal center dynamics for three different cocktails. The first two rows show cases for top two cocktails and the last row corresponds to the worst cocktail found during the combinatorial optimization search in Section 3.3. For every cocktail, the affinity maturation simulation over 21 days is repeated 5 times, and the average value of the quantity of interest is plotted with a margin of one standard deviation. The left column shows the progress of B-cell population size in the germinal center. The maturation of the B-cells toward the antigens in cocktail is shown in the middle column as an average value of affinities over all centrocytes and centroblasts in the germinal center. The count of unique antibody sequences (the right column) over the simulation period illustrates the sequence diversity in the germinal center.

We compute the structures of each antigen in the designed cocktail by selecting the top-ranked prediction from ColabFold (Mirdita *et al.* 2022). Lafit tool (Mann *et al.* 2012) discretizes these predicted protein chains and makes the corresponding 3D-lattice representation required for Ymir’s affinity model (Robert *et al.* 2021). Figure 4 illustrates the GC dynamics for three cocktails over the simulation period of 21 days after the dose of vaccine. For all three cocktails, the population of B-cell (the left column) progresses in typical response shown in Robert *et al.* (2021, 2024). They also show a similar trend in antibody diversity (right column). The key differences among these cocktails are in the trend of affinity maturation of the B-cells (middle column) to each antigen available in the cocktail. For the best cocktail (4,5,18), there is a clear hierarchy among the antigens, i.e. affinities to the second antigen (sequence 5) grows faster compared to the first antigen (sequence 4), and a similar relation between the first antigen and third antigen (sequence 18). However, for the second-best cocktail (3,4,20), the third antigen (sequence 20) dominates the whole dynamics which results in a very insignificant increase in affinity for the first antigen (sequence 3). This implies the latter antigen plays an insignificant role in the germinal dynamics. And for the worst cocktail (4,17,18)’s case, the B-cells show a similar increasing trend of affinities across all three antigens. If we compare this affinity dynamics with the case of the best cocktail, we observe that in the presence of sequence 5, affinity to sequence 4 is comparatively higher than sequence 18. But if we replace sequence 5 of the best cocktail with sequence 17, then the relative immunodominance level of sequences 4 and 18 is altered. This highlights the intricate relationship among the antigens of different immunogenicity levels which is not possible to capture with the sequence-based affinity maturation model we used for optimization. Moreover, such discrepancy between our optimization proxy objective $f_{\text{AM}}(x)$ and our desired goal of balanced and broad immune response, may guide our optimization strategy toward antigen cocktails which are more optimal for the proxy rather than the actual immunological outcome. For example, a hierarchical response similar to cocktail (4, 5, 18) can be problematic if the least immunogenic antigen is more important for some future strains. To make the optimization target more aligned with the goal of broad protection, one can adopt a regularized version of $f_{\text{AM}}(x)$ where high variance in immune response across the antigens of the cocktail is penalized. One could also utilize the observations from validation experiments in updating the knowledge of immunodominance hierarchy, and integrate it within the sequence-based simulation of $f_{\text{AM}}(x)$. However, further exploration is needed to determine whether such refinement approaches are compatible with the assumptions made within the *in silico* simulation tools.

## 4 Conclusion

In this work, we have formulated a screening and optimization-based framework for designing cocktail of antigens to illicit immunogenic response against potential new variants. Leveraging the DMS experimental data for SARS-CoV-2 WT’s RBD protein, our framework identified possible candidate antigens based on the predictions of ACE2-RBD binding affinity and antibody escape probability. To increase the diversity of antigens selected in the cocktail, we further narrowed down the pool of candidates based on the clusters derived from the antigen sequence embeddings given by a pLM. Experiments with different optimization strategies showed the advantages of Bayesian optimization over GA in designing optimal cocktails using efficacy feedback from a computational model of affinity maturation. In particular, we found that both strategies succeeded in outperforming sequential greedy optimization to find a better cocktail. Finally, we investigated for the presence of an imbalanced immune response in the designed cocktails by simulating a structure-based affinity maturation process.

The modular aspect of our approach—candidate antigen generation, affinity maturation guided search for optimal cocktail out of those candidates—eases the integration with other methodologies from vaccine design research to enhance its applicability to a different virus of interest. For example, we utilized predictions (e.g. ACE2-RBD2 binding, Ab escape probability) from neural networks to drive the antigen selection process where appropriate *in silico* and *in vivo* techniques can be integrated to enhance the screening efficiency. Moreover, the antigens are designed without explicitly considering the structure which is expected to be more informative about the binding activity. Incorporating structural information may increase the reliability of the designed cocktail. For example, one could potentially utilize antigen structure in the selection of single-mutation sites to narrow down the pool of single mutations (selected based on DMS data in our pipeline) to more structurally relevant mutations.

The current framework can be further extended by incorporating iterative feedback between its different components—candidate antigen generation and cocktail optimization. However, one needs to carefully consider the assumptions (e.g. representation of antigens, approximation of binding energy etc.) made in the affinity maturation simulation. Furthermore, the generation of antigens is initiated by selected single mutations. While this gives more control to the designers, it also creates a potential bias in the cocktail. The generative approach has the potential to be a more rational strategy but it primarily depends on the reliability of mutational effect prediction on the virus-receptor interaction (Chen *et al.* 2023).

## Supplementary Material

vbaf182_Supplementary_Data

## Data Availability

The code for cocktail design is available in https://github.com/nafizabeer/Antigen_Cocktail_Design. See the Supplementary Information, available as supplementary data at *Bioinformatics Advances* online for further details.
