# Supplementary material for: In silico design of immunogenic antigen cocktail via affinity maturation-guided optimization
Source: Bioinform Adv. 2025 Jul 28;5(1):vbaf182. doi: 10.1093/bioadv/vbaf182 (PMC12360842; doi:10.1093/bioadv/vbaf182)
Supplement: vbaf182_Supplementary_Data [file vbaf182_supplementary_data.pdf]

S1 Prediction of Antibody Escape

We use the proposed architecture of Wang (2023) to predict the antibody escape probability of an RBD sequence for an arbitrary antibody. This model first predicts the input embedding of the RBD sequence as well as the heavy and light chain of the antibody. A Transformer encoder processes these three input embeddings to make them a low dimensional sequence embedding. Three sequence embeddings are concatenated and transformed by a fully connected network to the predicted escape probability in the logarithm scale.

In Wang (2023), some residues of the RBD sequence are randomly masked during inference to make the prediction more robust to the sequence context. Additionally, mutated residues of the RBD and the set of escape residues (Greaney et al., 2021) for the class of the given antibody are never masked. We follow the same principle to predict the log escape probability of each RBD sequence for the 10 antibodies from Reincke et al. (2022). For each antibody, we predict the escape probability by running the prediction model on each randomly masked RBD sequence. We repeat the inference 100 times, and the average predictions are considered as Ab-escaping probability of the unmasked RBD sequence for that antibody. Finally, the average of these Ab-escaping probabilities for 10 antibodies is used to select the top 1% RBD sequences with high antibody escape probability.

We run inference on our designed antigen RBD sequences with the pre-trained model from Wang (2023). That model was trained with deep mutational scanning experiment data (Starr et al., 2020) for the WT sequence with different classes of antibodies. Since it was not trained for a specific antibody, it allows us to incorporate new antibodies in our design framework.

S2 Hyperparameter tuning of ACE2-RBD predictor

Based on the results demonstrated in Taft et al. (2022), we followed the ensemble of LSTM and Transformer architectures over simpler models for predicting binding of ACE2 with each RBM region. Specifically, we adopted the Transformer architecture over the random forest since the latter relies on the flattened one-hot encoding vector representations for the RBM sequence, resulting in the loss of sequential context. Table S1 shows the ranges of different hyperparameters searched using Optuna (Akiba et al., 2019) for each RBM specific ACE2 binding predictor. In Table S2, we have reported the specific hyperparameters for training the RBM-specific binding prediction networks which are applied in screening for ACE2-RBD binding sequences. Each network is trained with Adam optimizer (Kingma and Ba, 2014) for 20 epochs using the training split of the corresponding dataset. After each epoch, the trained model is applied to the validation split. The model with the best average precision score is retained as the trained model for screening application.

Table S1. Hyperparameters searched for each LSTM and Transformer based prediction network of RBD-ACE2.

Hyperparameter	Ranges
Learning rate	[1e-5, 1e-1]
Dropout probability	{0.1,0.2,0.3}
Dimension of hidden state in LSTM	{50,100,150}
Number of neurons in feedforward of Transformer	{120,160,200,240}
Number of LSTM layers	{1,2,3}
Number of layers in Transformer encoder	{1,2,3,4}

S3 Details of Bayesian Optimization

We have used Matérn kernel ($\nu = 5/2$) for the Gaussian process model f_{GP}. While the choice of kernel may affect the optimization performance, we did not analyze this in our work.

The probabilistic reparameterization trick (Daulton et al., 2022) allows us to maximize the acquisition function $\alpha(\mathbf{x})$ by optimizing the continuous variable $\boldsymbol{\theta}$ which governs $p(\mathbf{x}|\boldsymbol{\theta})$. Specifically, for the continuous parameters $\boldsymbol{\theta} \in [0,1]^N$.

Table S2. Specific values of hyperparameters in the RBM specific ACE2 binding prediction networks used for screening

Network	Hyperparameter	RBM1-ACE2	RBM2-ACE2	RBM3-ACE2
LSTM	Learning rate	2e-2	3e-3	4e-3
	Dropout probability	0.2	0.2	0.1
	Dimension of hidden state in LSTM	50	150	50
	Number of LSTM layers	4	2	3
Transformer	Learning rate	2e-3	1e-3	1e-3
	Dropout probability	0.1	0.1	0.1
	Number of neurons in feedforward of Transformer	120	120	120
	Number of layers in Transformer encoder	2	3	3

$p(\mathbf{x}|\boldsymbol{\theta})$ is an N- dimensional Bernoulli distribution, where each ith dimension follows a Bernoulli distribution with success probability θ_i. To sample the representation of our cocktail, i.e. $\mathbf{x} \in \{0,1\}^N$ from $p(\mathbf{x}|\boldsymbol{\theta})$, we assign each x_i to 1 or 0 (i^{th} antigen being present in the cocktail $\mathbf{x}$ or not) with probability θ_i and $1-\theta_i$ respectively.

S4 Data and code availability

- DMS data for WT in Starr et al. (2020), https://github.com/jbloomlab/SARS-CoV-2-RBD_DMS/tree/master/results
- Training data of ACE2-RBD binding from Taft et al. (2022)
- Ab escape predictor model, and test antibody data from Wang (2023), https://github.com/ericzwang/RBD_AB

S4.1 Affinity maturation simulation

In the cocktail optimization stage, we have used the sequence-based affinity maturation model from Wang and Chakraborty (2022) available in https://github.com/ericzwang/sars2-vaccine.

For the structure-based affinity maturation simulation Robert et al. (2021, 2024) used in the validation, the program is available in https://gitlab.com/Sporistos/Microvima. We ran the germinal center simulation for our designed cocktails based on the parameter files shared by the authors of Robert et al. (2024).

S4.2 Cocktail optimization

The codes for cocktail design are available in https://github.com/nafizabeer/Antigen_Cocktail_Design.

References

Akiba, T., Sano, S., Yanase, T., Ohta, T., and Koyama, M. (2019). Optuna: A next-generation hyperparameter optimization framework. In *Proceedings of the 25th ACM SIGKDD International Conference on Knowledge Discovery and Data Mining*.

Daulton, S., Wan, X., Eriksson, D., Balandat, M., Osborne, M. A., and Bakshy, E. (2022). Bayesian optimization over discrete and mixed spaces via probabilistic reparameterization. *Advances in Neural Information Processing Systems*.

Greaney, A. J., Starr, T. N., Barnes, C. O., Weisblum, Y., Schmidt, F., Caskey, M., Gaebler, C., Cho, A., Agudelo, M., Finkin, S., et al. (2021). Mapping mutations to the sars-cov-2 rbd that escape binding by different classes of antibodies. *Nature communications*.

Kingma, D. P. and Ba, J. (2014). Adam: A method for stochastic optimization. *arXiv preprint arXiv:1412.6980*.

Reincke, S. M., Yuan, M., Kornau, H.-C., Corman, V. M., van Hoof, S., Sánchez-Sendin, E., Ramberger, M., Yu, W., Hua, Y., Tien, H., et al. (2022). Sars-cov-2 beta variant infection elicits potent lineage-specific and cross-reactive antibodies. *Science*.

Robert, P. A., Arulraj, T., and Meyer-Hermann, M. (2021). Ymir: A 3d structural affinity model for multi-epitope vaccine simulations. *Iscience*.

Robert, P. A., Arulraj, T., and Meyer-Hermann, M. (2024). Germinal centers are permissive to subdominant antibody responses. *Frontiers in Immunology*.

Starr, T. N., Greaney, A. J., Hilton, S. K., Ellis, D., Crawford, K. H., Dingens, A. S., Navarro, M. J., Bowen, J. E., Tortorici, M. A., Walls, A. C., et al. (2020). Deep mutational scanning of sars-cov-2 receptor binding domain reveals constraints on folding and ace2 binding. *Cell*.

Taft, J. M., Weber, C. R., Gao, B., Ehling, R. A., Han, J., Frei, L., Metcalfe, S. W., Overath, M. D., Yermanos, A., Kelton, W., et al. (2022). Deep mutational learning predicts ace2 binding and antibody escape to combinatorial mutations in the sars-cov-2 receptor-binding domain. *Cell*.

Wang, E. (2023). Prediction of antibody binding to sars-cov-2 rbds. *Bioinformatics Advances*.

Wang, E. and Chakraborty, A. K. (2022). Design of immunogens for eliciting antibody responses that may protect against sars-cov-2 variants. *PLOS Computational Biology*.
